# Molecular mechanisms and potential therapeutic targets in the pathogenesis of hypertension in visceral adipose tissue induced by a high-fat diet

**DOI:** 10.3389/fcvm.2024.1380906

**Published:** 2024-04-16

**Authors:** Zhenyang Su, Jin-Yu Sun, Min Gao, Wei Sun, Xiangqing Kong

**Affiliations:** ^1^ School of Medicine, Southeast University, Nanjing, China; ^2^ Department of Cardiology, The First Affiliated Hospital With Nanjing Medical University, Nanjing, China

**Keywords:** hypertension, high-fat diet, visceral adipose tissue, pathogenicity factors, therapeutic target

## Abstract

**Background:**

Hypertension (HTN) presents a significant global public health challenge with diverse causative factors. The accumulation of visceral adipose tissue (VAT) due to a high-fat diet (HFD) is an independent risk factor for HTN. While various studies have explored pathogenic mechanisms, a comprehensive understanding of impact of VAT on blood pressure necessitates bioinformatics analysis.

**Methods:**

Datasets GSE214618 and GSE188336 were acquired from the Gene Expression Omnibus and analyzed to identify shared differentially expressed genes between HFD-VAT and HTN-VAT. Gene Ontology enrichment and protein-protein interaction analyses were conducted, leading to the identification of hub genes. We performed molecular validation of hub genes using RT-qPCR, Western-blotting and immunofluorescence staining. Furthermore, immune infiltration analysis using CIBERSORTx was performed.

**Results:**

This study indicated that the predominant characteristic of VAT in HTN was related to energy metabolism. The red functional module was enriched in pathways associated with mitochondrial oxidative respiration and ATP metabolism processes. Spp1, Postn, and Gpnmb in VAT were identified as hub genes on the pathogenic mechanism of HTN. Proteins encoded by these hub genes were closely associated with the target organs-specifically, the resistance artery, aorta, and heart tissue. After treatment with empagliflozin, there was a tendency for Spp1, Postn, and Gpnmb to decrease in VAT. Immune infiltration analysis confirmed that inflammation and immune response may not be the main mechanisms by which visceral adiposity contributes to HTN.

**Conclusions:**

Our study pinpointed the crucial causative factor of HTN in VAT following HFD. Spp1, Postn, and Gpnmb in VAT acted as hub genes that promote elevated blood pressure and can be targets for HTN treatment. These findings contributed to therapeutic strategies and prognostic markers for HTN.

## 1 Introduction

Globally, cardiovascular disease persists as the foremost cause of death. In spite of the global reduction in cardiovascular mortality, there is a discernible increase in the overall number of fatalities. Among these cardiovascular conditions, hypertension (HTN), particularly elevated systolic blood pressure, stands out as the primary contributor to the cardiovascular burden (1, 2). The multifaceted pathogenesis of HTN encompasses intricate interactions among genetic, environmental, and lifestyle factors. Recent research has progressively directed attention towards the influence of modified visceral adipose tissue (VAT) resulting from a high-fat diet (HFD) regimen in the onset of HTN (3–6). The comprehension of the underlying molecular mechanisms and signaling pathways intrinsic to this complex association is paramount for the formulation of precise interventions and strategies for prevention.

The consumption of HFD, often associated with modern sedentary lifestyles and dietary habits, has been identified as a significant causative factor in the obesity epidemic (7, 8). The excessive accumulation of VAT, a hallmark of obesity, is known to induce chronic low-grade inflammation and disrupt metabolic homeostasis (3, 9, 10). This alteration in adipose tissue function is intricately linked to the development of various metabolic disorders, prominently including HTN (11). VAT possesses the capacity to release various inflammatory factors, including tumor necrosis factor-alpha (TNF-α) and interleukin-6 (IL-6), which may lead to endothelial cell damage, lipid deposition, and fibrosis in the arteries, thereby affecting vascular tone and blood pressure regulation (12, 13).

Moreover, VAT is more active in releasing fatty acids and hormones (e.g., epinephrine and insulin inhibitory factor) that directly impact blood pressure regulation compared to subcutaneous adipose tissue (SAT) (14, 15). Insulin resistance is also one of the mechanisms through which abdominal obesity contributes to increased blood pressure. Insulin resistance causes the body to require more insulin to maintain normal blood glucose levels, but the vasodilatory effect of insulin on blood vessels is diminished, potentially leading to increased blood pressure (16, 17). Additionally, chemical signals released by visceral adipose tissue can stimulate the sympathetic nervous system, resulting in an elevated heart rate and blood pressure (18).

While substantial evidence suggests that HFD-induced accumulation of VAT is an independent risk factor for HTN, its primary impact on blood pressure appears to be mediated through the influence on glycolipid metabolism and perivascular adipose tissue's (PVAT) release of vasoactive substances (12, 18). There is currently no evidence supporting the assertion that inflammation of visceral adipose tissue, a component of the systemic chronic inflammation observed in obesity, significantly contributes to the development of HTN. Hence, the specific causative factor in VAT for HTN remains unclear. Conducting bioinformatics analyses to explore the effects of VAT alterations on HTN requires a thorough examination of the intricate network of molecular interactions within VAT and its connections with other physiological systems. The advancement of high-throughput technologies and computational tools has opened unprecedented opportunities to dissect the complex transcriptomic landscapes associated with HTN related to obesity. This study aims to unravel specific alterations in gene expression, signaling pathways, and molecular networks within VAT induced by a HFD regimen. Bioinformatics methodologies, such as differentially expressed gene (DEG) analysis, pathway enrichment analysis, protein-protein interaction (PPI) network analysis and immune infiltration analysis, will be employed to identify key molecular players and reveal the regulatory cascades pivotal in the progression from VAT to HTN.

## 2 Materials and methods

### 2.1 Data collection and processing

Raw expression profiling datasets, GSE214618 for VAT transcriptome sequencing in mice subjected to HFD and those on a chow diet, and GSE188336 for VAT transcriptome sequencing in Spontaneously Hypertensive Rats (SHR) and normotensive Wistar-Kyoto (WKY) rats, were sourced from the National Center for Biotechnology Information Gene Expression Omnibus (GEO, https://www.ncbi.nlm.nih.gov/gds/) (19). Additionally, expression profiling datasets of resistance arteries (GSE8051), aortas (GSE145854), heart tissues (GSE207283) and kidney tissues (GSE66494) from SHR and WKY rats were also obtained from the GEO database. The transcriptome expression profiling dataset GSE206986 of multiple tissues from treated SHR was downloaded for validation of the relationship between hub genes and the efficacy of treatment. Detailed descriptive information on datasets was shown in Table 1. All data analyzed in Fragments per Kilobase of Transcript per Million Mapped Reads (FPKM) format. The processing and analysis of data were conducted using the R software, and the limma package in R was utilized for data normalization (20).

**Table 1:** Descriptive statistics of the GEO datasets.

GEO accession	Species	Origin	Sample	
** **	** **	** **	Chow	HFD
GSE214618	Mus musculus	VAT	6	7
** **		** **	WKY	SHR
GSE188336	Rattus norvegicus	VAT	6	6
GSE8051	Rattus norvegicus	Resistance artery	3	3
GSE145854	Rattus norvegicus	Aorta	3	3
GSE207283	Rattus norvegicus	Heart tissue	3	3
GSE225146	Rattus norvegicus	Kidney tissue	6	6
** **	** **	** **	SHR	SHR with empagliflozin
GSE206986	Rattus norvegicus	Aorta, Brown fat, Brain, Kidney, Liver, Lung, White fat	3	3

### 2.2 Identification of DEGs

The limma package in R was used for the analysis of DEGs. The screening criteria for DEGs were set as |log2 Fold Change| ≥ 1 and *p*-value < 0.05. Visualization of DEGs was accomplished by constructing volcano plots using the ggplot2 package in R. Heatmaps for sample clustering were generated using the heatmap package in R. Venn diagrams illustrating the overlapping sets of DEGs among HFD-VAT and HTN-VAT were created using EVenn (https://www.ehbio.com/test/venn/#/) (21).

### 2.3 Functional enrichment analysis

Metascape (https://metascape.org/gp/index.html#/) as an online resource for gene annotation and analysis, was used to perform Gene Ontology (GO) enrichment analysis of DEGs (22). A significance threshold of *p* < 0.05 was employed to determine statistical significance. The top 20 enriched pathways identified in the analysis were presented.

### 2.4 PPI analysis

The PPI analysis of DEGs was performed utilizing the Search Tool for the Retrieval of Interacting Genes (STRING) database (https://cn.string-db.org/) (23). Subsequently, the PPI network was exported and constructed using Cytoscape software (version.3.9.1, Institute for Systems Biology, US) (24). Sub-network modules were discerned using the molecular complex detection (MCODE) plugin (25). The criteria for determining biologically significant complexes were set as an MCODE score >3 and a node number >3. Genes located within these modules were designated as hub genes. Additionally, the Maximal Clique Centrality (MCC) algorithm in the cytoHubba plugin was also used to identify hub genes with high connectivity in PPI networks (26).

### 2.5 Immune infiltration analysis

To assess the similarities and distinctions in the immune microenvironment within HFD-VAT and HTN-VAT, discrepancies in immune infiltration were analyzed between the HFD and chow groups, as well as between the SHR and WKY groups. This analysis was performed using CIBERSORTx (https://cibersortx.stanford.edu/). The outcomes were then visualized through box plots.

### 2.6 Animals

Sixteen-week-old SHR and WKY rats were sourced from Vital River Laboratories (Beijing, China) and maintained on a standard chow diet. Four-week-old Sprague-Dawley rats, also sourced from Vital River Laboratories, were subjected to a diet comprising either regular chow or high-fat chow (high transfat (44 kcal%) high cholesterol (2%) high fructose (22%), #AMLN, Dyets, US) for a duration of 12 weeks. Epididymal white adipose tissues were excised for subsequent molecular verification. All animal experimental procedures adhered to the guidelines and received approval from the Institutional Animal Care and Use Committee of Nanjing Medical University (Approval No. IACUC-1906038).

### 2.7 Real-time quantitative polymerase chain reaction (RT-qPCR)

RNA extraction was conducted using RNAiso Plus (#9109, TAKARA, Japan), following the manufacturer's instruction. The reverse transcriptase reactions were conducted using the PrimeScriptTM RT Reagent Kit (#RR047A, Takara, Japan). The Hieff UNICON® qPCR SYBR Green Master Mix (#11184ES03, Yeasen, China) was used for PCR system. The program was executed on a Real-time PCR instrument (ABI, US). The relative expression levels of targeted mRNAs and miRNAs were calculated using the 2^−*ΔΔ*Ct^ method and normalized the values to β-actin. The detailed primer sequences are as follows: Spp1 (Forward primer: TGTCCTCTGAAGAAACGGAT, Reverse primer: ATCATCGTCCATGTGGTCA), Postn (Forward primer: TCGTGGAACCAAAAATTAAAGTC, Reverse primer: CTTCGTCATTGCAGGTCCTT), Gpnmb (Forward primer: TCCTGGTGGATGGGACTAGG, Reverse primer: CCCCCAAACTCCAGTCAAGG), and β-actin (Forward primer: CCTCACTGTCCACCTTCCA, Reverse primer: GGGTGTAAAACGCAGCTCA).

### 2.8 Western blotting

Total proteins were extracted using RIPA lysis buffer (#P0013B, Beyotime, China) containing 1mM phenylmethanesulfonyl fluoride (#ST506, Beyotime, China). Protein quantification was performed with the Enhanced BCA Protein Assay Kit (#P0010, Beyotime, China). After adding SDS-PAGE loading buffer (#P0015, Beyotime, China), protein samples were heated in a metal bath at 99 °C for 10 min. Subsequently, the samples were analyzed via Western blotting on 10% SDS-PAGE and transferred onto polyvinylidene difluoride membranes (Millipore, USA). Following a 20 min blocking step with protein-free rapid blocking buffer (#G2052, Servicebio, China), the membrane was incubated overnight at 4 °C with primary antibodies for Spp1 (1:1000, #AF7665, Beyotime, China), Postn (1:1000, #AF7792, Beyotime, China), Gpnmb (1:1000, #GB111475, Servicebio, China), and β-actin (1:1000, #AF0003, Beyotime, China). Subsequently, the membrane was incubated with HRP-linked secondary antibodies (#FDM007/FDR007, FDbio, China) for 2 h at room temperature. The blots were visualized using a ChemiDoc MP imager (BioRad, US).

### 2.9 Immunofluorescence staining

For immunofluorescence staining, the paraffin-embedded adipose tissue samples underwent dewaxing, rehydration, and antigen retrieval. Non-specific binding sites were sealed using 10% goat serum. Then, the samples were incubated overnight at 4 °C with anti-Spp1 (#AF7665, Beyotime, China), anti-Postn (#AF7792, Beyotime, China) and anti-Gpnmb (#bs-2684R, Bioss, China), followed by incubation with Alexa Fluor 488 goat anti-rabbit IgG (H + L) (#111-007-003, Jackson, US) for 1 h at room temperature. The samples were sealed with a DAPI-containing sealing solution (#P0131, Beyotime, China). Finally, the samples were observed under a fluorescence microscope (Zeiss, Germany).

### 2.10 Statistics

All statistical analyses were performed using GraphPad Prism (version.9.0, GraphPad Software, US). The data were presented as mean ± SD. To assess statistical differences between two groups, an unpaired Student's *t*-test was employed. A *p*-value < 0.05 was considered statistically significant.

## 3 Results

### 3.1 Data processing

**Figure 1:**
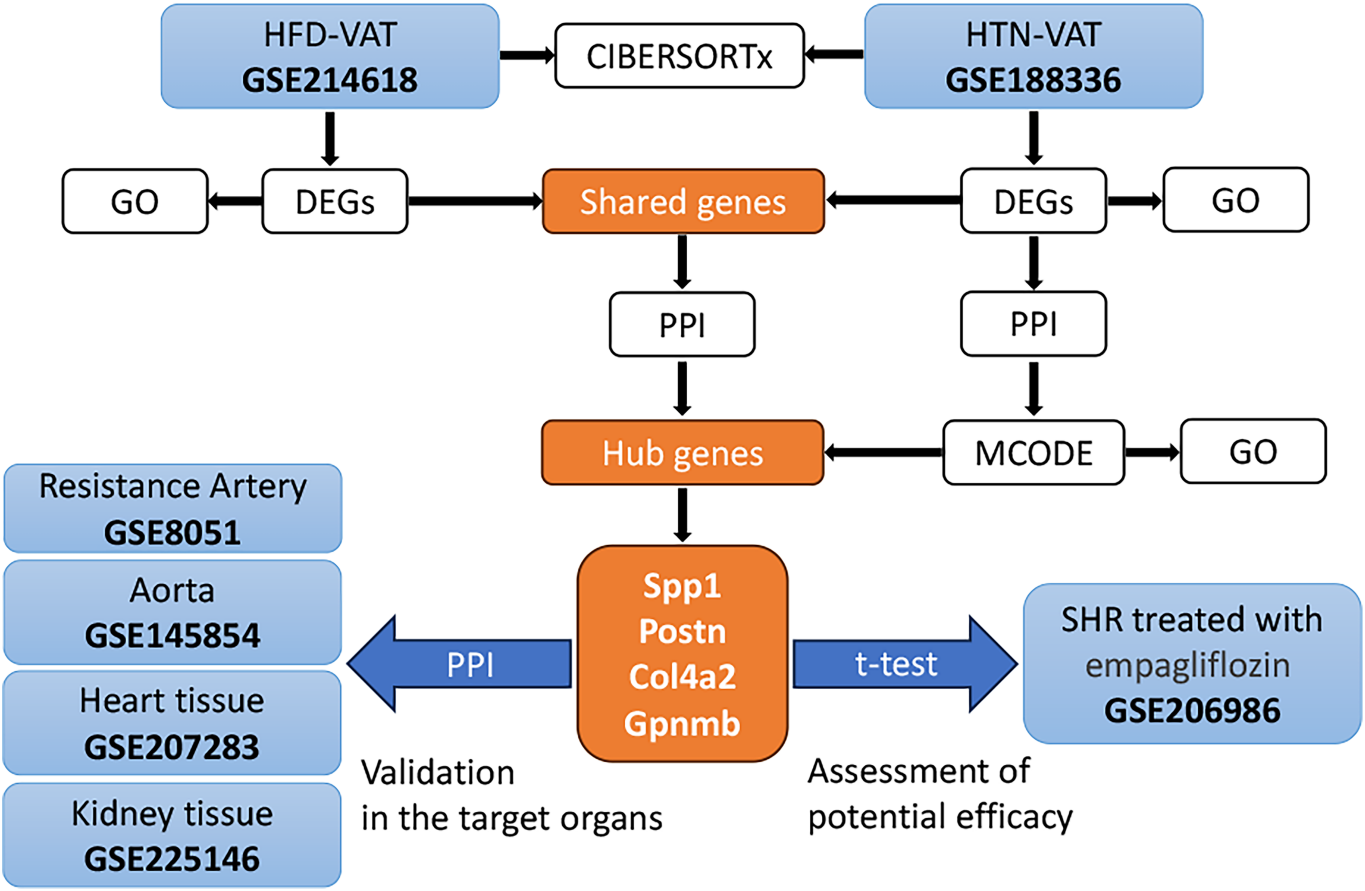
The strategy of bioinformatics analysis for this study.

The strategy of bioinformatics analysis was performed as shown in Figure 1. Initially, the identification and analysis of DEGs were conducted to assess GO enrichment for VAT in HFD compared to controls, as well as for VAT in HTN vs. controls. Subsequently, PPI analysis was executed on the up-regulated DEGs common to both groups. Additionally, PPI analysis was independently performed on the DEGs within the HTN dataset, and hub genes were derived from the combined analysis. The pivotal role of these hub genes was confirmed across diverse tissue samples, and alterations in hub genes were scrutinized following treatment for HTN. Lastly, immune infiltration analysis was carried out to compare immune cell infiltration in VAT post HFD with that observed in HTN.

### 3.2 Identification of DEGs in HFD-VAT and enrichment analysis

**Figure 2:**
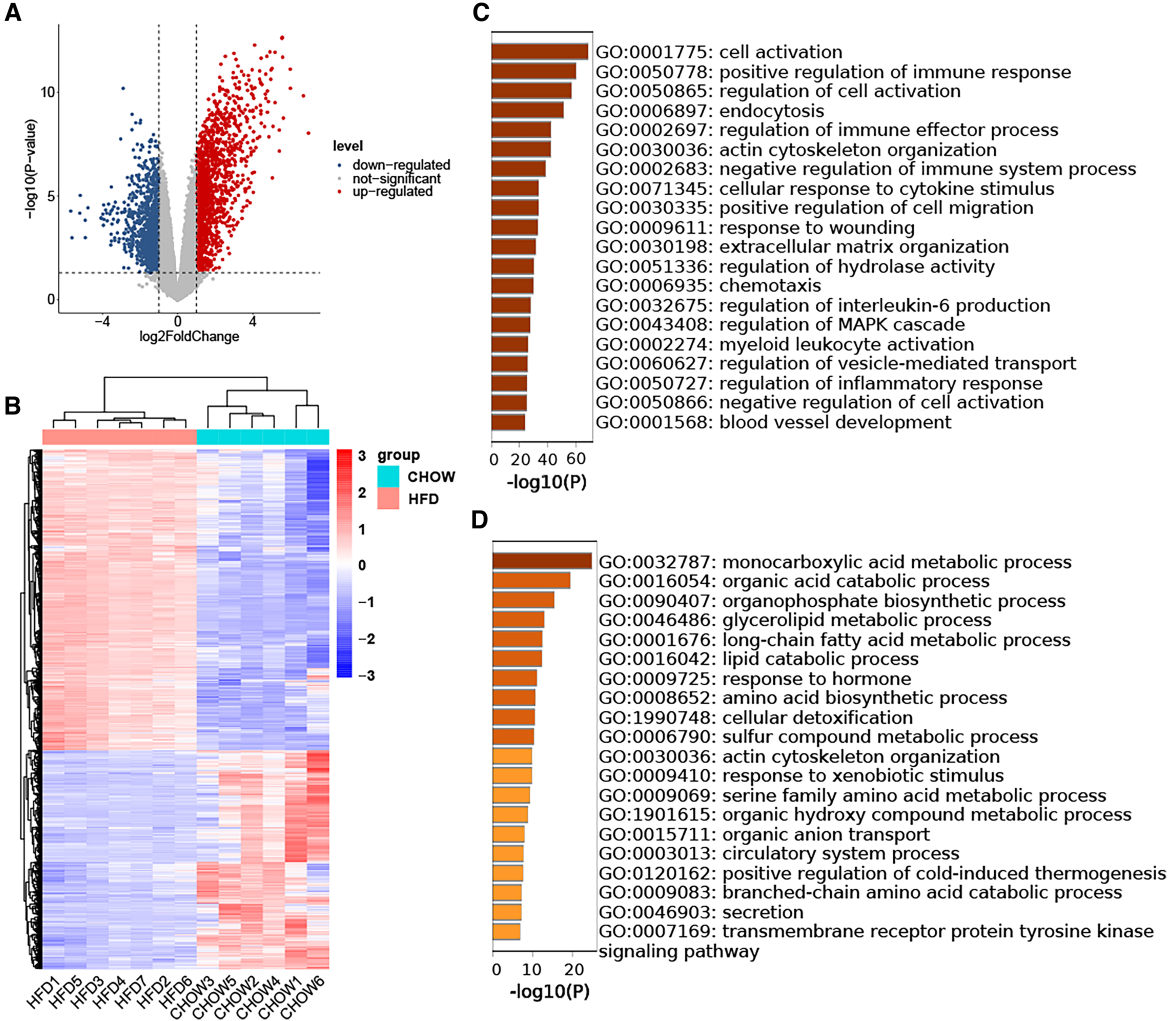
Identification of DEGs in HFD-VAT and enrichment analysis. (**A**) Volcano plot depicting the distribution of DEGs. (**B**) Heatmap and group clustering of DEGs. (**C**) GO-BP enrichment analysis of up-regulated DEGs. (**D**) GO-BP enrichment analysis of down-regulated DEGs.

The GSE214618 dataset was utilized to analyze the DEGs in VAT between the HFD and chow diet groups. The dataset underwent meticulous pre-processing to generate an expression matrix encompassing 14,776 genes, devoid of any missing values. Employing a significance threshold of *p*-value < 0.05 and |log2 Fold Change| ≥ 1, a total of 1,349 up-regulated DEGs and 978 down-regulated DEGs were identified between the HFD and chow diet groups. The distribution of these DEGs is visually depicted in the volcano plot (Figure 2A). Subsequent clustering of samples based on the DEGs distinctly differentiated the HFD group from the control group (Figure 2B). GO enrichment analysis was conducted separately for up-regulated DEGs and down-regulated DEGs, focusing on Biological Processes (BP). The results showed that the up-regulated genes were enriched in cell activation, regulation of immune response, endocytosis, actin cytoskeleton organization, cellular response to cytokine stimulus, regulation of cell migration, response to wounding, extracellular matrix organization, regulation of hydrolase activity, chemotaxis, regulation of interleukin-6 production, regulation of MAPK cascade, myeloid leukocyte activation, regulation of vesicle-mediated transport, regulation of inflammatory response, blood vessel development (Figure 2C). The down-regulated genes were mainly enriched in biosynthetic and metabolic processes of endogenous substances such as fatty acids and amino acids (Figure 2D).

### 3.3 Identification of DEGs in HTN-VAT and enrichment analysis

**Figure 3:**
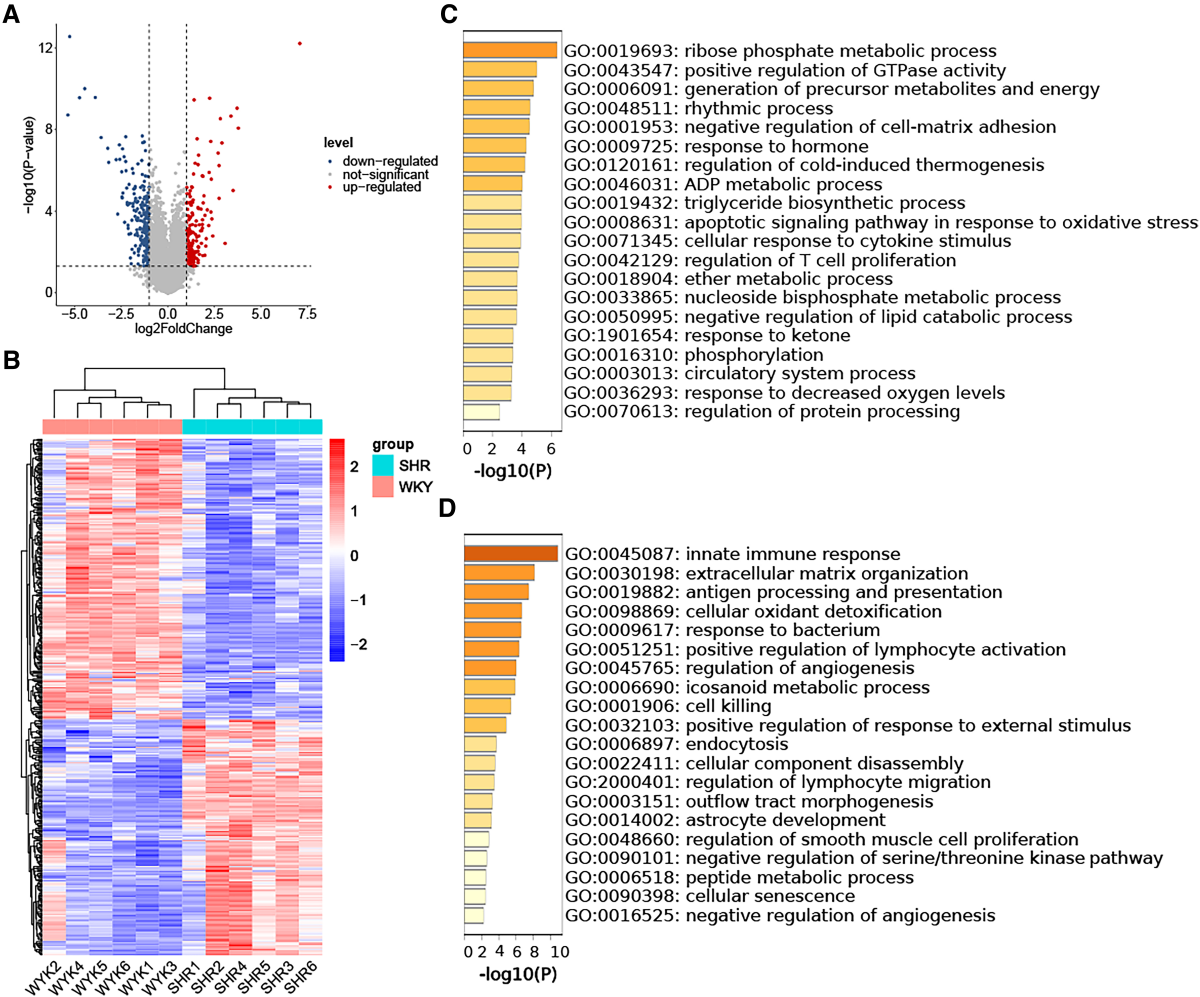
Identification of DEGs in HTN-VAT and enrichment analysis. (**A**) Volcano plot depicting the distribution of DEGs. (**B**) Heatmap and group clustering of DEGs. (**C**) GO-BP enrichment analysis of up-regulated DEGs. (**D**) GO-BP enrichment analysis of down-regulated DEGs.

The analysis of DEGs in VAT between the SHR and WKY groups utilized the GSE188336 dataset. Following preprocessing, an expression matrix encompassing 32,883 genes with no missing values was generated. Ultimately, 161 up-regulated DEGs and 172 down-regulated DEGs were identified, employing a significance threshold of *p*-value < 0.05 and |log2 Fold Change| ≥ 1. Volcano plot and heatmap analyses were employed to illustrate the expression patterns of DEGs in the HTN dataset (Figures 3A,B). The results of the GO-BP enrichment analysis showed that the up-regulated genes were enriched in ribose phosphate metabolic process, positive regulation of GTPase activity, generation of precursor metabolites and energy, rhythmic process, negative regulation of cell-matrix adhesion, response to hormone, regulation of cold-induced thermogenesis, ADP metabolic process, triglyceride biosynthetic process, apoptotic signaling pathway in response to oxidative stress, cellular response to cytokine stimulus, regulation of T cell proliferation, ether metabolic process, nucleoside bisphosphate metabolic process, negative regulation of lipid catabolic process, response to ketone, phosphorylation, circulatory system process, response to decreased oxygen levels, regulation of protein processing (Figure 3C). The down-regulated genes enriched to the GO-BP pathways included innate immune response, extracellular matrix organization, antigen processing and presentation, cellular oxidant detoxification, response to bacterium, positive regulation of lymphocyte activation, regulation of angiogenesis, icosanoid metabolic process, cell killing, positive regulation of response to external stimulus, endocytosis, cellular component disassembly, regulation of lymphocyte migration, outflow tract morphogenesis, astrocyte development, regulation of smooth muscle cell proliferation, negative regulation of serine/threonine kinase pathway, peptide metabolic process, cellular senescence, negative regulation of angiogenesis (Figure 3D).

### 3.4 Shared DEGs for VAT in HTN and in HFD

**Figure 4:**
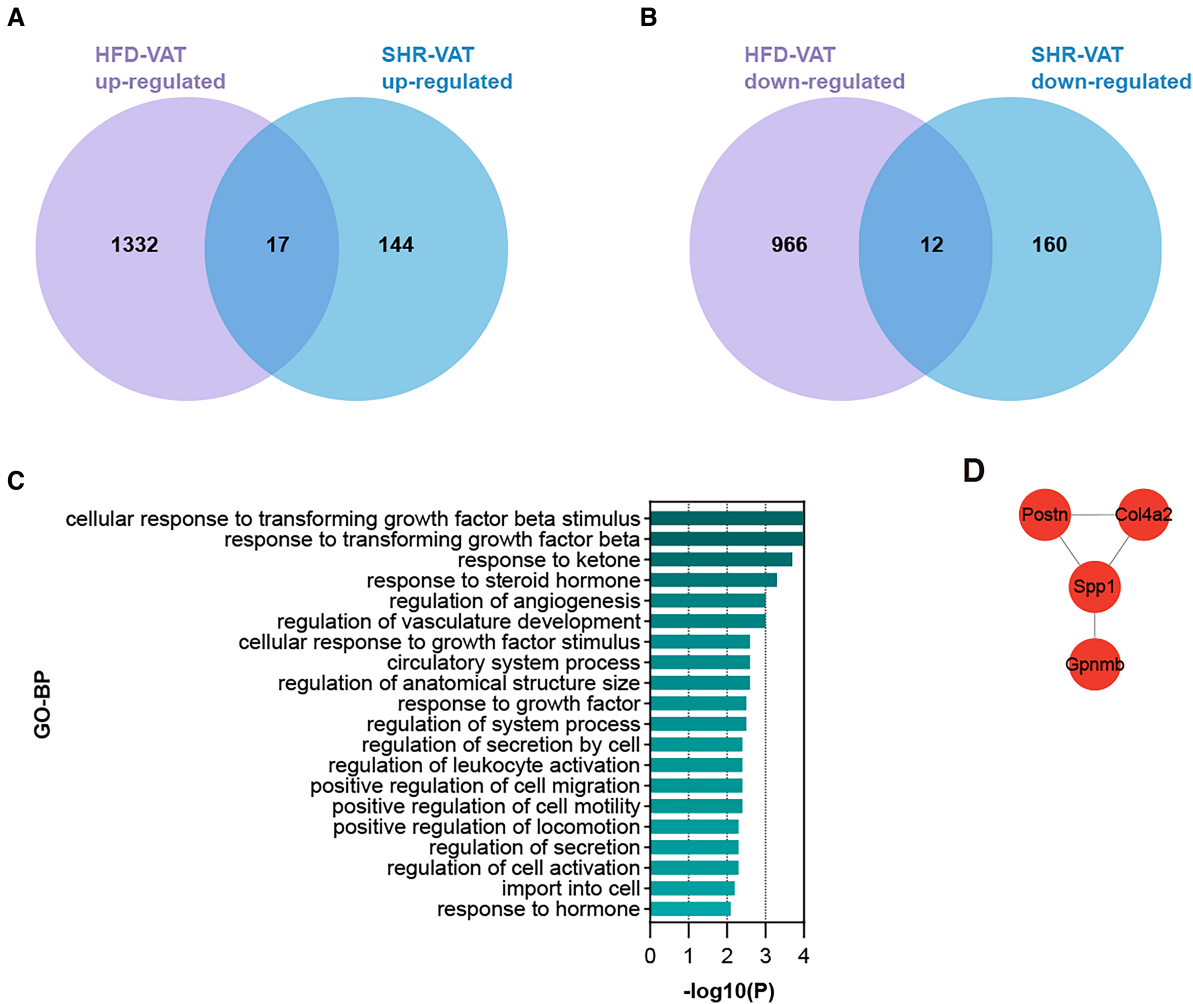
Shared DEGs for VAT in HTN and in HFD. (**A**) Overlap of up-regulated DEGs in the HFD dataset and the HTN dataset. (**B**) Overlap of down-regulated DEGs in the HFD dataset and the HTN dataset. (**C**) GO-BP enrichment analysis of the shared up-regulated DEGs. (**D**) PPI analysis of the shared up-regulated DEGs.

To identify DEGs shared by VAT in the two pathologic states, Venn diagrams were used to reveal 17 up-regulated and 12 down-regulated overlapping genes respectively (Figures 4A,B). Enrichment analysis revealed that the shared up-regulated DEGs were primarily associated with the GO-BP pathways, including cellular response to transforming growth factor beta stimulus, response to ketone, response to steroid hormone, regulation of angiogenesis, regulation of vasculature development, cellular response to growth factor stimulus, circulatory system process, regulation of anatomical structure size, regulation of system process, regulation of secretion by cell, regulation of activation, positive regulation of cell migration, positive regulation of cell motility, positive regulation of locomotion, import into cell, response to hormone (Figure 4C). PPI analysis of the shared up-regulated DEGs was conducted, identifying Spp1, Postn, Col4a2, and Gpnmb as core genes within the constructed interaction network (Figure 4D).

### 3.5 Identification of functional modules and hub genes in the DEGs of HTN-VAT

**Figure 5:**
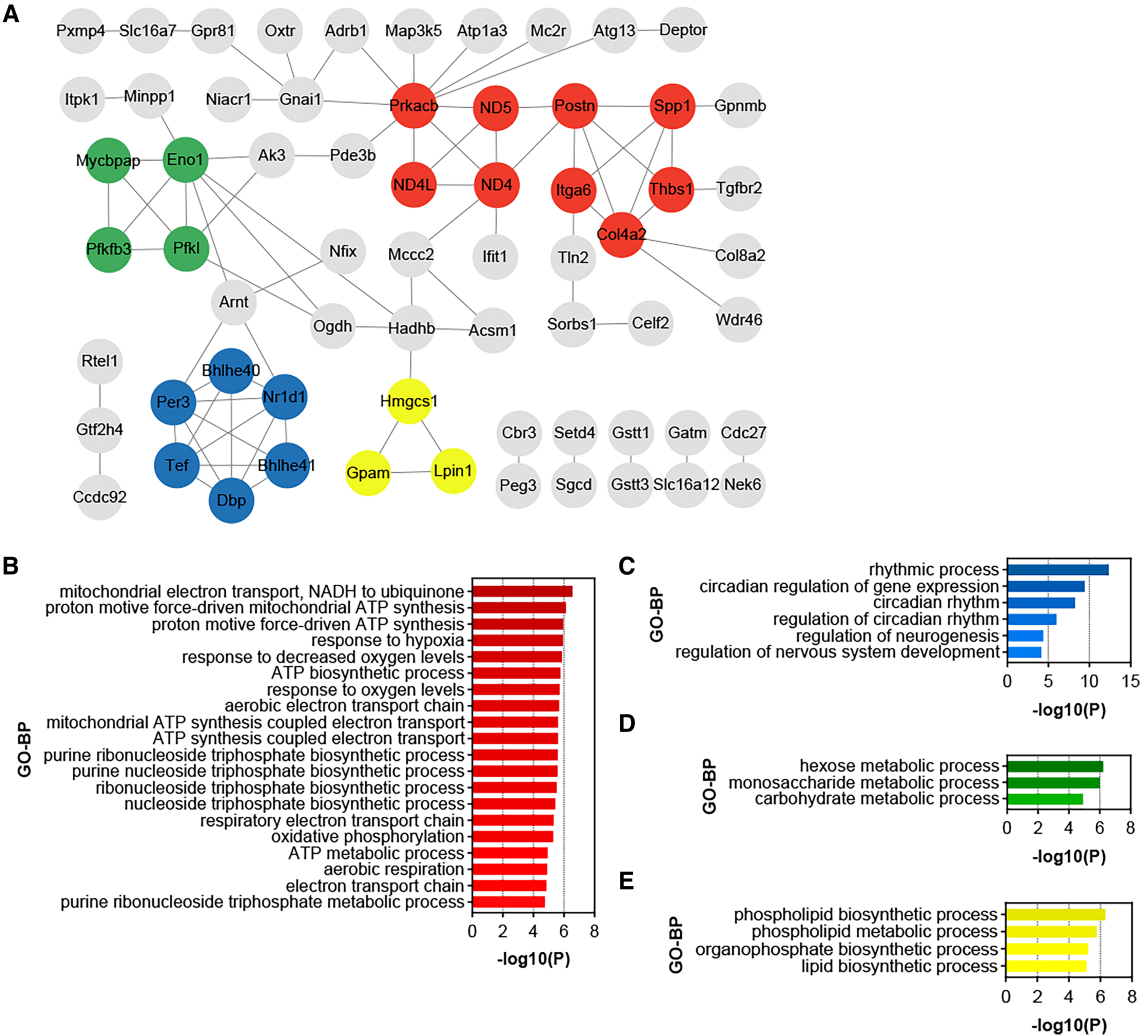
Identification of functional modules and hub genes in the DEGs of HTN-VAT. (**A**) PPI analysis of the up-regulated DEGs in HTN-VAT. (**B**) GO-BP enrichment analysis of the genes in the red module. (**C**) GO-BP enrichment analysis of the genes in the blue module. (**D**) GO-BP enrichment analysis of the genes in the green module. (**E**) GO-BP enrichment analysis of the genes in the yellow module.

PPI analysis was conducted on all up-regulated DEGs within the GSE188336 dataset. Functional modules within the PPI network were identified using the MCODE algorithm, with four distinctive functional modules highlighted (Figure 5A). The red module encompasses Spp1, Postn, Itga6, Thbs1, Col4a2, ND5, ND4, ND4l, and Prkacb. Among these, Spp1, Postn, and Col4a2 also feature as hub genes shared with HFD-VAT (Figure 4D). The red module was enriched to GO-BP pathways mainly including mitochondrial electron transport, ATP biosynthetic process, response to hypoxia, oxidative phosphorylation, aerobic respiration (Figure 5B). The blue, green and yellow functional modules were then mainly enriched in regulation of circadian rhythm, monosaccharide metabolic process and phospholipid biosynthetic process, respectively (Figures 5C-E).

### 3.6 Role of hub genes in target organs

**Figure 6:**
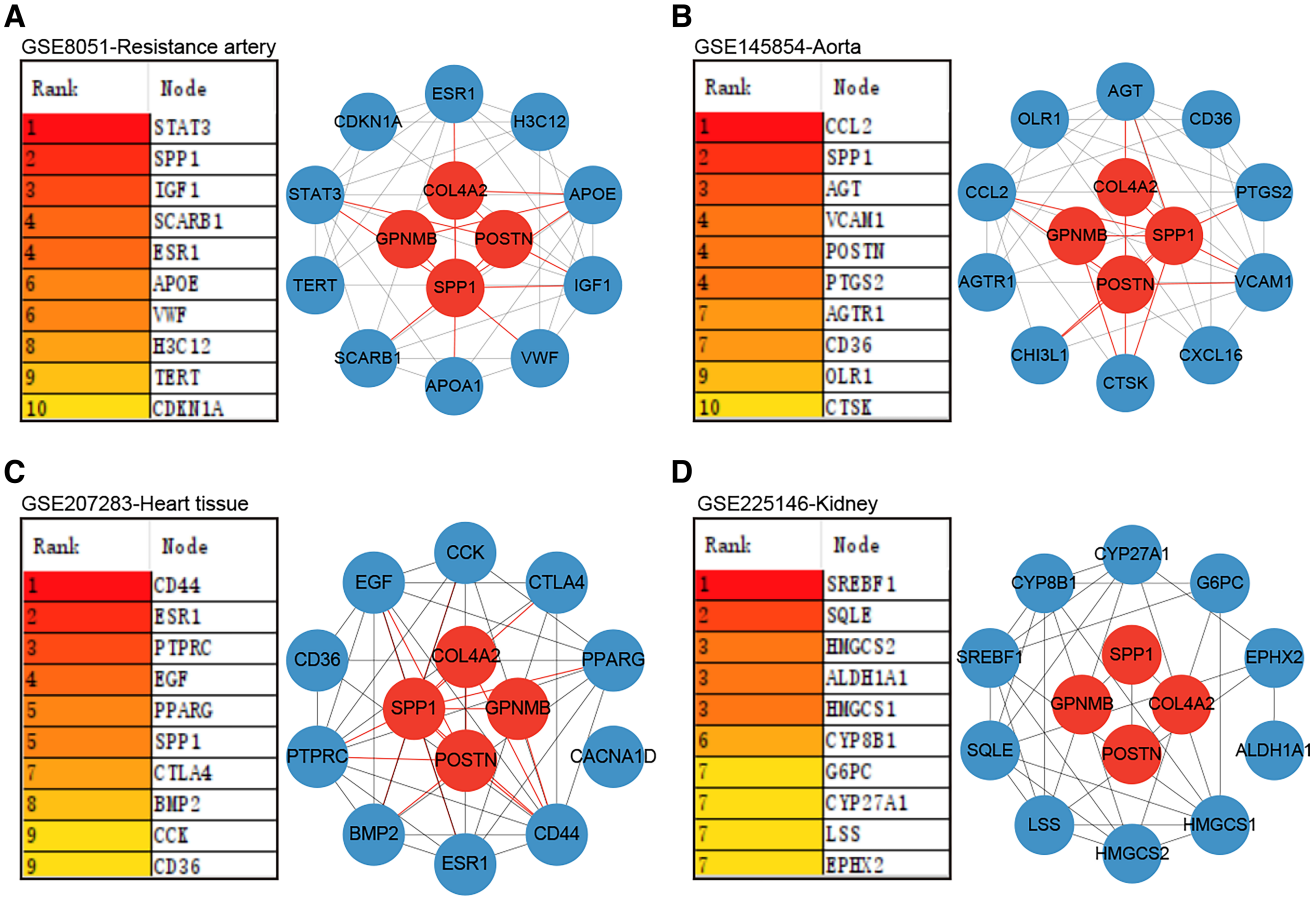
Role of hub genes in target organs. (**A**) PPI analysis of hub genes in dataset GSE8051 and importance ranking by MCC algorithm. (**B**) PPI analysis of hub genes in dataset GSE145854 and importance ranking by MCC algorithm. (**C**) PPI analysis of hub genes in dataset GSE207283 and importance ranking by MCC algorithm. (**D**) PPI analysis of hub genes in dataset GSE225146 and importance ranking by MCC algorithm.

Proteins synthesized and secreted by adipose tissue exert effects on gene expression in other organs through the circulation. The impacts of hub genes and the proteins they encode on hypertensive target organs were further analyzed using datasets GSE8051, GSE145854, GSE207283, and GSE225146, which incorporate transcriptome sequencing results from the resistance artery, aorta, heart tissue, and kidney tissue of SHR and WKY rats, respectively. Notably, Spp1, Postn, Col4a2, and Gpnmb were not identified as DEGs using a significance threshold of *p*-value < 0.05 and |log2 Fold Change| ≥ 1. Consequently, these four hub genes were additionally included in the PPI analyses with the DEGs in each dataset. In the resistance artery dataset, Spp1 ranked second in the PPI analyses, and the hub genes closely interacted with proteins from the top ten ranked DEGs (Figure 6A). Within the aortic dataset, Spp1 and Postn ranked second and fourth, respectively, in the PPI analysis, with the hub genes interacting closely with proteins from the top ten DEGs (Figure 6B). In the heart tissue dataset, Spp1 ranked fifth in the PPI analysis, and the hub gene interacted closely with proteins from the top 10 DEGs (Figure 6C). Conversely, poor protein interactions were observed between hub genes and DEGs in the renal dataset (Figure 6D).

### 3.7 Therapeutic potential of hub genes

**Figure 7:**
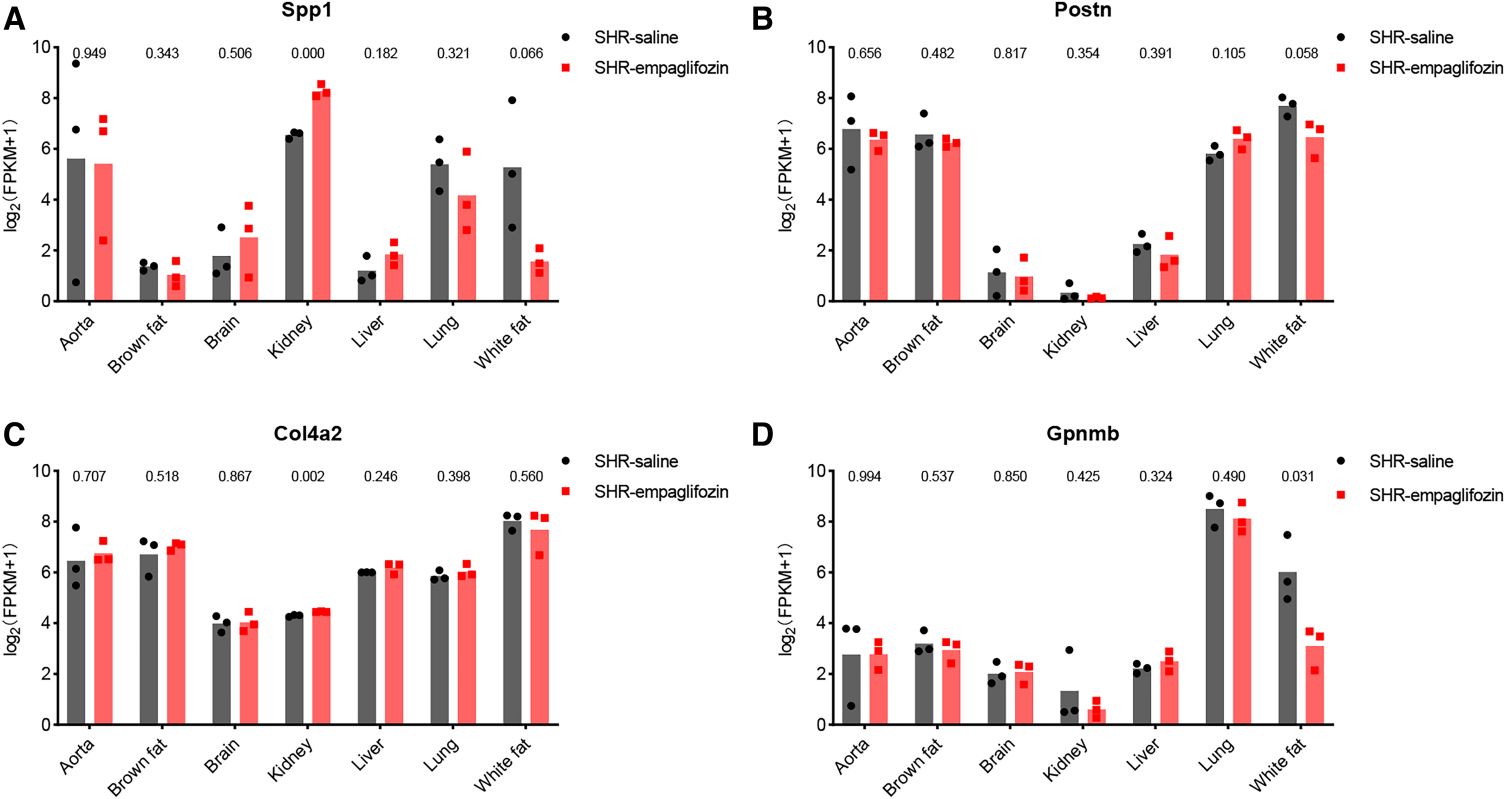
Therapeutic potential of hub genes. (**A**) Changes in the expression of Spp1 in various tissues after treatment with empagliflozin. (**B**) Changes in the expression of Postn in various tissues after treatment with empagliflozin. (**C**) Changes in the expression of Col4a2 in various tissues after treatment with empagliflozin. (**D**) Changes in the expression of Gpnmb in various tissues after treatment with empagliflozin.

Changes in central genes in various organs of SHR rats treated with empagliflozin were analyzed using the dataset GSE206986, especially changes in hub genes in white fat. Despite not achieving statistical significance (*p* = 0.066), the observed change in Spp1 in white fat was notably substantial (Figure 7A). Following treatment with empagliflozin, Spp1 expression experienced a significant decrease in white fat from SHR. Similarly, Postn exhibited a reduction in white fat in the treatment group, although the statistical evidence was not robust (*p* = 0.058) (Figure 7B). Col4a2 expression remained unaffected by empagliflozin (Figure 7C). Remarkably, the expression of Gpnmb in white fat significantly decreased after empagliflozin treatment (*p* = 0.031) (Figure 7D).

### 3.8 Validation of hub genes

**Figure 8:**
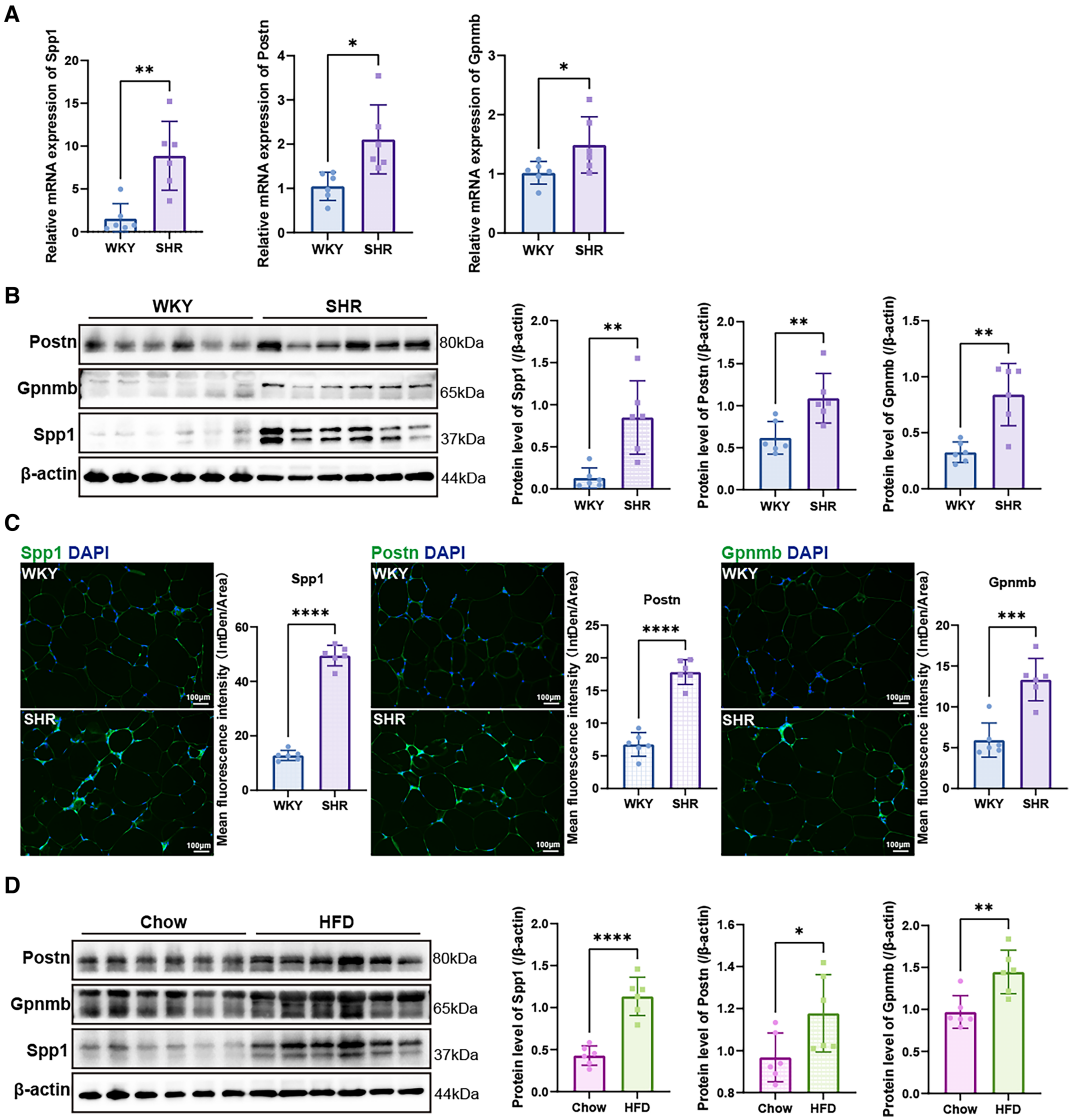
Validation of hub genes. (**A**) RT-qPCR was used to validate the mRNA levels of Spp1, Postn and Gpnmb in epididymal white adipose tissue of WKY and SHR (*n* = 6). (**B**) Protein expression levels of Spp1, Postn, and Gpnmb in epididymal white adipose tissue of WKY and SHR were presented by Western blotting (*n* = 6). (**C**) Immunofluorescence staining showed the expression and distribution of Spp1, Postn and Gpnmb in epididymal white adipose tissue of WKY and SHR (*n* = 6). (**D**) Protein levels of Spp1, Postn, and Gpnmb were confirmed through Western blotting analysis in the epididymal white adipose tissue of rats subjected to diets of either regular chow or high-fat chow (*n* = 6). Results were presented with mean ± SD (one-way ANOVA followed by Bonferroni *post hoc* tests, *p < 0.05, **p < 0.01, ***p < 0.001, ****p < 0.0001).

The mRNA expression levels of the hub genes, namely Spp1, Postn, and Gpnmb, exhibited a notable increase in SHR compared to WKY rats in the context of VAT (*p* < 0.05) (Figure 8A). Western blotting and immunofluorescence analyses further confirmed a significant elevation in the expression of the proteins encoded by these three genes in SHR (*p* < 0.05) (Figures 8B,C). Furthermore, the expression of Spp1, Postn, and Gpnmb was corroborated to be elevated in VAT of rats subjected to a high-fat diet (Figure 8D).

### 3.9 Immune infiltration analysis

**Figure 9:**
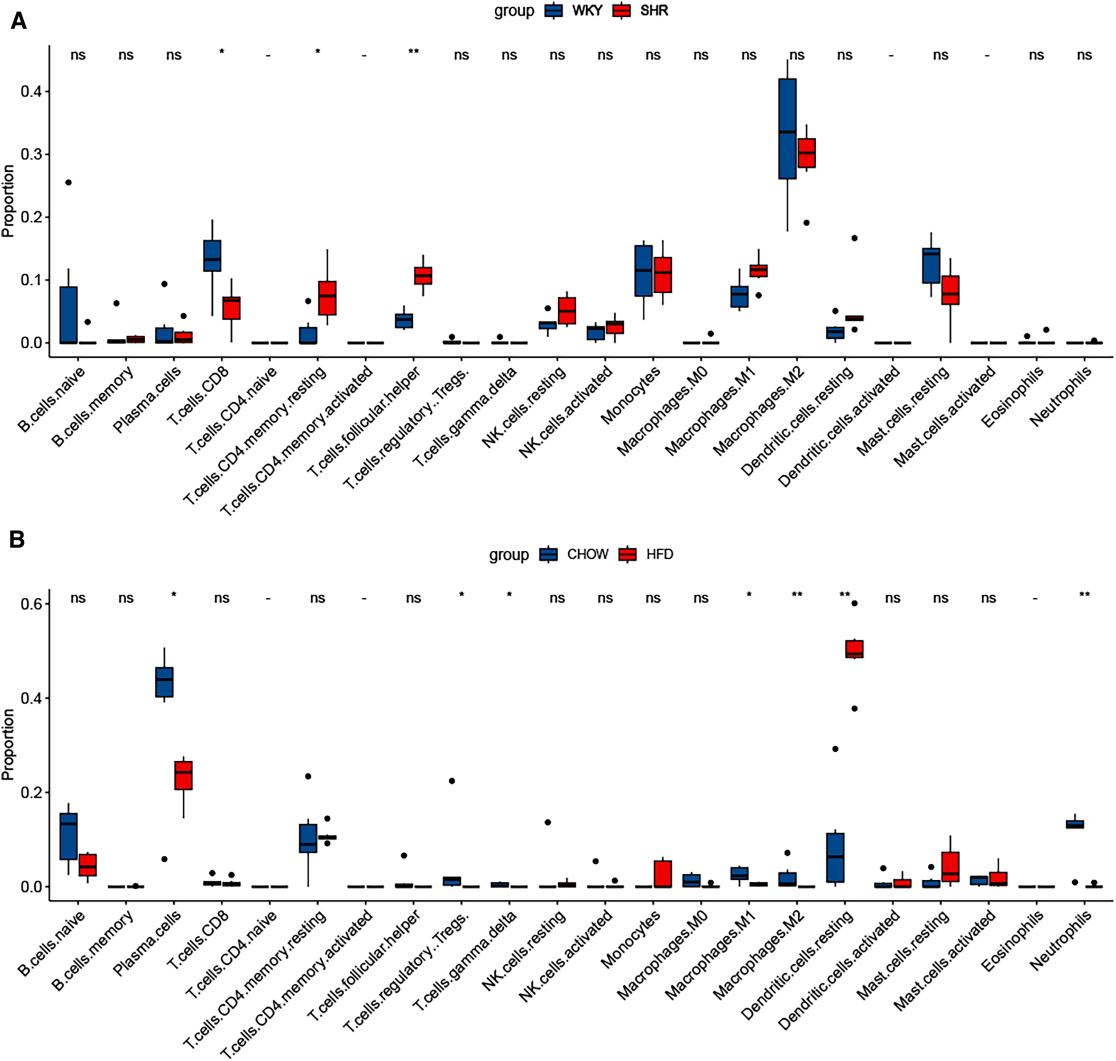
Immune infiltration analysis. (**A**) Immune infiltration analysis of the dataset GSE188336. (**B**) Immune infiltration analysis of the dataset GSE214618.

The characterization of immune cells was determined by CIBERSORTx. In comparison to the WKY group, SHR-VAT displayed higher proportions of T cells CD4 memory resting and T cells follicular helper, whereas lower proportions of *T* cells CD8 (Figure 9A). Compared to the chow diet group, HFD-VAT demonstrated increased proportions of Dendritic cells resting, accompanied by decreased proportions of Plasma cells, *T* cells regulatory Tregs, *T* cells gamma delta, Macrophages M1, Macrophages M2 and Neutrophils (Figure 9B).

## 4 Discussion

A substantial body of evidence indicates a robust link between the accumulation of adipose tissue (AT) across the body and the emergence of cardiovascular diseases (27). The distribution of AT plays a crucial role in determining the risk of cardiovascular disease, with elevated VAT associated with a higher prevalence of cardiovascular metabolic disorders, including HTN, dyslipidemia, and insulin resistance, compared to SVT (14, 15, 28). PVAT represents a distinct category of VAT responsible for modulating vascular tone through the release of vasodilatory mediators, including nitric oxide, adiponectin, and angiotensin 1–7, in physiological circumstances (18, 29–31). Conversely, in the context of obesity and insulin resistance, the heightened expression of angiotensin II and the escalated inflammatory response within PVAT contribute to compromised vasodilation and increased vascular stiffness, ultimately culminating in elevated blood pressure (32, 33). However, the deposition of VAT induced by obesity not only increases the amount of PVAT, but non-perivascular VAT including retroperitoneal AT and rodent epididymal AT increases even more. It remains important to explore new genes, potential diagnostic biomarkers, and prospective therapeutic targets associated with the development of HTN in VAT through comprehensive bioinformatics analysis.

This study reiterated that cell activation, inflammation, and immune response were the primary features of VAT with HFD, as indicated by the enrichment analysis of DEGs, consistent with prior research (34, 35). In contrast, the predominant characteristic of VAT in HTN appeared to be related to energy metabolism. Correspondingly, the enrichment analysis of shared up-regulated DEGs highlighted the response of VAT to endogenous substances, such as transforming growth factor beta. Among the functional modules derived from protein interactions, the red module was enriched in pathways associated with mitochondrial oxidative respiration and ATP metabolism processes. This suggested that, in addition to the inflammatory response, other molecular mechanisms within VAT may contribute to the promotion of HTN. In essence, the onset of HTN appeared to be more associated with changes in energy metabolic pathways within VAT than with inflammation in VAT. Significantly, while adipose inflammation may not emerge as a predominant factor in the pathogenesis of HTN, indications of the inflammatory response were still evident in the VAT of SHR. This was underscored by the enrichment of pathways regulating *T*-cell proliferation. Moreover, immune infiltration analysis revealed substantial *T*-cell infiltration in the VAT of SHR.

In this study, Spp1 was identified as the most hub gene contributing to HTN in VAT following HFD. Spp1, also known as Osteopontin (Opn), has been mentioned in several studies for its association with the development and progression of HTN. Spp1 is significantly elevated in the peripheral blood of hypertensive patients (36, 37). Overexpression of Spp1 leads to vascular inflammation, endothelial cell proliferation and migration, and ultimately promotes atherosclerosis as well as exacerbates vascular fibrosis and remodeling (38–43). The pathogenic mechanism of Spp1-induced HTN may also be related to impaired renal function. Several studies have suggested that aberrant expression of bone-bridging proteins may be associated with renal tubular dysfunction, which in turn affects blood pressure regulation (44–46). Expression of spp1 in cardiac tissue is also upregulated in HTN and further exacerbates cardiac fibrosis and dysfunction (47–51). However, the *in situ* role of spp1 may not be a major contributor to pathogenesis. Our study revealed a significant upregulation of Spp1 expression in both VAT with HFD and VAT associated with HTN. Spp1 has been identified as a hub gene in VAT closely linked to the development of HTN. Consequently, with the accumulation of VAT, spp1, with its elevated expression level, could be a pivotal factor in the initiation and progression of HTN. It may exert its influence on multiple target organs, including small arteries, aortas, and heart tissue, thereby exacerbating the deterioration of blood pressure. Spp1 in VAT may emerge as a promising therapeutic target for hypertension, deserving further investigation.

In addition to Spp1, Postn and Gpnmb were also emerged as contributors to the impact of VAT on blood pressure during HFD. Postn is a secreted extracellular matrix protein like Spp1. Previous research has suggested that Postn may play a role in aortic thickening in mice with DOCA salt-induced HTN (52). Additionally, metformin has been shown to mitigate hyperlipidemia-associated vascular calcification by downregulating Postn (53). However, existing reports have primarily focused on elevated Postn in vascular *in situ*. Indeed, similar to Spp1, it is Postn released from VAT that may be pivotal to pathogenicity. Gpnmb, or Glycoprotein non-metastatic melanoma protein B, also known as Osteoactivin, is a glycoprotein implicated in various biological processes such as immune regulation and certain aspects of tumor development (54, 55). However, no studies have confirmed its association with the development of HTN. This study has validated that the proteins encoded by all three of these hub genes were closely associated with the target organs-specifically, the resistance artery, aorta, and heart tissue.

In this study, we not only confirmed that Spp1, Postn and Gpnmb were key causative factors of HTN in VAT after HFD, but also validated the prognostic role of these key genes after treatment using the dataset GSE206986. This dataset includes transcriptome expression profiles of multiple tissues from SHR rats treated with a sodium-glucose cotransporter 2 (SGLT2) inhibitor empagliflozin. Although HTN is not an indication for engeletin, the treatment remained effective (56). Throughout the blood pressure-lowering course, there was a tendency for Spp1, Postn, and Gpnmb to decrease in visceral white adipose tissue, with insignificant changes in Col4a2. This suggested that the decline of these three hub genes in VAT may be a crucial mechanism for SGLT2 inhibitor-induced blood pressure reduction. It further validated that Spp1, Postn, and Gpnmb were key factors in the elevation of blood pressure due to the accumulation of VAT.

Finally, we conducted immune infiltration analysis of HFD-VAT and HTN-VAT, respectively. The results showed distinct infiltration phenotypes of immune cells between the two conditions. *T* cells CD4 memory resting and *T* cells follicular helper were significantly elevated in SHR compared to WKY, and there was no significant difference in Macrophages.M2, which accounted for the majority. In contrast, the proportion of Dendritic cells resting was significantly elevated in HFD-VAT compared with those on a chow diet, consistent with findings from previous studies (57–59). In conclusion, both immune infiltration analysis and enrichment analysis of DEGs confirmed that, although inflammation and immune responses were involved, they may not constitute the primary mechanism through which VAT contributes to HTN.

The limitations of this study should be acknowledged. All analyses were conducted through a bioinformatics approach, and further validation through animal experiments is essential to confirm the hypertensive effects of Spp1, Postn, and Gpnmb in VAT. Fully validating the conclusions of this study would necessitate a series of intricate experiments, including interventions to manipulate the expression of hub genes to observe their impact on blood pressure. Additionally, investigating whether antihypertensive drugs could exert their effects by inhibiting these hub genes is essential. Previous research has demonstrated the effects of SGLT2 inhibitors on hub genes, prompting the exploration of whether other medications such as GLP-1 could similarly influence these genes and effectively treat HTN. Furthermore, further investigation into the role of disrupted energy metabolism in VAT in promoting blood pressure is warranted.

## 5 Conclusion

Our study pinpointed the crucial causative factor of HTN in VAT following HFD. Inflammatory activation and immune response in VAT did not appear to be major contributors to the pathogenesis. Enrichment analysis of VAT revealed that the major pathways in the pathogenesis of HTN were response to endogenous substances, mitochondrial oxidative respiration, and ATP metabolism processes. Spp1, Postn, and Gpnmb in VAT acted as hub genes and had a key influence on the mechanism of HTN. Inhibition of the expression of these genes in VAT may play an important role in the treatment of HTN. These findings contributed to therapeutic strategies and prognostic markers for HTN.

## Data Availability

The transcriptome data used in this study were sourced from the National Center for Biotechnology Information Gene Expression Omnibus (GEO, https://www.ncbi.nlm.nih.gov/gds/), including the datasets GSE214618, GSE188336, GSE8051, GSE145854, GSE207283, GSE66494 and GSE206986. Other data supporting the results of this study are available upon reasonable request from the corresponding author.
